# Getah virus triggers ROS-mediated autophagy in mouse Leydig cells

**DOI:** 10.3389/fmicb.2024.1519694

**Published:** 2025-01-13

**Authors:** Fengqin Li, Lishuang Deng, Tong Xu, Lei Xu, Zhiwen Xu, Siyuan Lai, Yanru Ai, Yanqun Wang, Guangwen Yan, Ling Zhu

**Affiliations:** ^1^College of Veterinary Medicine, Sichuan Agricultural University, Chengdu, China; ^2^College of Animal Science, Xichang University, Xichang, China; ^3^Key Laboratory of Animal Disease Detection and Prevention in Panxi District, Xichang University, Xichang, China; ^4^Key Laboratory of Animal Diseases and Human Health of Sichuan Province, Sichuan Agricultural University, Chengdu, China

**Keywords:** Getah virus, ROS, autophagy, mouse, Leydig cells

## Abstract

**Introduction:**

Getah virus (GETV) is a zoonotic virus transmitted via a mosquito-vertebrate cycle. While previous studies have explored the epidemiology and pathogenicity of GETV in various species, its molecular mechanisms remain largely unexplored.

**Methods:**

This study investigated the impact of GETV infection and associated molecular mechanisms on reactive oxygen species (ROS) and autophagy levels in mouse Leydig cells both *in vivo* and *in vitro*. The male mice and TM3 cells were treatment with N-acetylcysteine (NAC) to reduce cellular ROS levels. Rapamycin (Rapa) and 3-Methyladenine (3- MA) were used to change autophagy in both infected and uninfected TM3 cells.

**Results and Discussion:**

The findings revealed that GETV infection in mouse testes speciffcally targeted Leydig cells and induced oxidative stress while enhancing autophagy in testicular tissue. Using TM3 cells as an *in vitro* model, the study confirmed GETV replication in this cell line, triggering increased ROS and autophagy levels. Treatment with N-acetylcysteine (NAC) to reduce cellular ROS levels markedly reduced autophagy in testicular tissue and TM3 cells infected with GETV. Interestingly, the use of rapamycin (Rapa) and 3-Methyladenine (3- MA) led to autophagy change in both infected and uninfected TM3 cells, with no signiffcant alterations in cellular ROS levels. These results indicate that GETV infection elevates ROS levels, subsequently inducing autophagy in mouse Leydig cells. We also found that autophagy plays an important role in GETV replication. When autophagy levels were reduced using NAC and 3-MA, a corresponding decrease in TCID_50_ was observed. Conversely, upregulation of autophagy using Rapa resulted in an increase in TCID_50_ of GETV. Therefore, we speculate that GETV may exploit the autophagy pathway to facilitate its replication. These ffndings illuminate the interplay between GETV and host cells, providing valuable insights for therapeutic strategies targeting autophagy in GETV infections.

## 1 Introduction

Getah virus (GETV) belongs to the genus Alphavirus of the family Togaviridae, causing infections in humans and animals (Zhang et al., 2021). The virus maintains a natural transmission cycle among mosquitoes, vertebrate hosts, and environmental reservoirs (Li B. et al., 2022). Since its first isolation from Malaysian mosquitoes in 1955, GETV has been widely reported in island nations throughout the South Pacific (Li B. et al., 2022; Li et al., 2017b). Additionally, GETV infections have also been reported in various regions and animal species in China (Shi et al., 2022; Liu et al., 2023). The virus remains infectious under diverse environmental conditions, facilitating its transmission to susceptible hosts (Zhai et al., 2024). The geographic range and host species of GETV continue to expand. Global climate warming, which increases the range, duration, and frequency of mosquito activity, may contribute to the expansion of the virus’s ecological niche. As a mosquito-borne zoonotic virus, GETV poses serious threats to both livestock farming and public health (Sam et al., 2022). Further research on the pathogenicity and mechanisms of GETV is necessary to mitigate the potential threat of rapid and widespread transmission of the virus in the context of climate change.

The impact of GETV on the reproductive function of male animals has been confirmed, resulting in a reduction in offspring number, testicular damage, and a decline in both sperm quality and quantity (Li F. et al., 2022). Our initial research also revealed that following infection, GETV colonizes the testes of male mice exclusively through the Leydig cells of testicular tissue (Li F. et al., 2022). In mice, Leydig cells cluster between seminiferous tubules, serving as the primary source of testosterone and playing essential roles in metabolism, development, immunity, and sperm production (Aladamat and Tadi, 2024; Hu et al., 2023). TM3 cells, derived from Leydig cells of 11- to 13-day-old male mice, retain the physiological functions of Leydig cells *in vitro* and are extensively used in scientific research (Zhao et al., 2022). Therefore, TM3 cells provide a suitable in vitro model to study the effects of GETV infection on male reproductive capacity. To date, no studies have reported GETV infection in TM3 cells or elucidated the underlying mechanisms in vitro.

Various viral infections have been shown to induce oxidative stress, which impairs the normal function of host cells (Zhang et al., 2019). The accumulation of reactive oxygen species (ROS) is a major contributor to oxidative stress (Jomova et al., 2023). Leydig cells are rich in mitochondria and serve as a structural site for ROS production (Monageng et al., 2023). ROS act as oxidants and mediators of cellular damage, disease, homeostasis, and signal activation. Once the balance between ROS accumulation and ROS clearance is disrupted, oxidative stress occurs (Jakubczyk et al., 2020). Many positive-sense RNA viruses replicate in the ER–mitochondria contact membrane, inducing the unfolded protein response (UPR) that leads to the synthesis and reorganization of membranes, many of which involve oxidative stress (Gullberg et al., 2015; Lee and Ou, 2024). Because viral infections cause cellular stress, autophagy is often induced as a cellular response to infection (Ashkenazi and Dixit, 1998; Chen et al., 2023; Li et al., 2024).

Autophagy represents a fundamental mechanism of cell fate regulation. Autophagy plays a vital role in maintaining good health by removing old or damaged cells (Klionsky et al., 2021). Conversely, autophagy is also implicated in the onset and progression of various conditions caused by pathogenic infections (Deretic, 2021). Numerous studies have indicated that ROS can act as cell signaling molecules, initiating the formation and degradation of autophagosomes while regulating intracellular autophagy signaling (Wang et al., 2023). The interaction between ROS and autophagy is complex and takes place under diverse pathological conditions (Li et al., 2015; Filomeni et al., 2015). The autophagy induced by Chikungunya virus (CHIKV), which belongs to the Alphavirus genus along with GETV, is mediated by independent induction through the endoplasmic reticulum and oxidative stress pathways. It delays apoptotic cell death via ROS-mediated mTOR inhibition (Joubert et al., 2012). However, the changes in ROS levels and autophagy in Leydig cells following GETV infection and their interactions are not yet fully understood.

This study aimed to evaluate changes in oxidative stress and autophagy in the Leydig cells of GETV-infected mice and to further explore their underlying mechanisms. It provides insights into the mechanisms by which GETV infection affects male reproductive function, revealing that GETV triggers oxidative stress-mediated autophagy in Leydig cells. Understanding these mechanisms is particularly relevant given the expanding ecological range of GETV, driven by climate change, and the significant risks it poses to public health and livestock. These findings provide a foundation for future research on the effects of viral infections on male fertility and may guide the development of therapeutic strategies to mitigate reproductive damage caused by GETV.

## 2 Materials and methods

### 2.1 Animals, cells and virus

Five-week-old male specific-pathogen-free Kunming mice, weighing 30–35 g, were obtained from Chengdu Dossy Experimental Animals Co., Ltd. All experimental procedures involving animals were approved by the Sichuan Agricultural University Animal Ethical and Welfare Committee (approval number 20230152). The mice were acclimated for approximately 1 week before the experiment. They were housed under standard conditions, including a 12 h reversed light/dark cycle, a constant temperature of 22 ± 2°C, and 70 ± 4% humidity, with access to standard feed and water ad libitum.

After acclimatization, the mice were randomly assigned to two experimental groups. Twenty-four mice were further divided into four groups. The mice in the control group received 100 μl of DMEM as a vehicle to maintain consistency in the experimental conditions. In the GETV group, male mice were intramuscularly injected with 100 × TCID₅₀ in 0.1 ml of GETV at the posterior limb. Mice in the NAC group were intraperitoneally injected with 100 mg/kg N-acetylcysteine (NAC; an ROS inhibitor) based on body weight (Turkmen et al., 2019). Mice in the NAC-GETV group received the same acetylcysteine dosage 1 h after GETV injection. At 24 h post-infection (hpi), the mice were euthanized using CO₂, and their testes were harvested for further analysis.

TM3 cells were obtained from the American Type Culture Collection (ATCC). The cells were cultured in Dulbecco’s Modified Eagle’s Medium F-12 (DMEM/F-12; Gibco, 11320033), supplemented with 5% horse serum (Gibco, 16050122) and 2.5% fetal calf serum (FBS; Gibco, 12484028), and maintained in a 5% CO₂ incubator at 37°C. The dosage of NAC, Rapa and 3-MA were selected based on previous studies. NAC was used at a concentration of 0.5 mM (Ren et al., 2020), while Rapa was used at 100 nM (Wang P. et al., 2022), and 3-MA was used at 10 mM (Li J. et al., 2023).

The GETV-SC201807 strain (GenBank accession No. MK693225.1) was isolated from an aborted sow on a pig farm in Sichuan Province, China, and was preserved by the Animal Biology Technology Center, Sichuan Agricultural University (Chengdu, China; Jiang, 2021). Detect the viral TCID_50_ of GETV in TM3 cells and testicular tissue following the method described by Lei (Lei et al., 2021).

### 2.2 Immunohistochemical staining

Paraffin blocks of testicular tissues were subjected to immunohistochemical (IHC) assays using a SABC immunohistochemical staining kit (BOSTER, SA1020). A rabbit polyclonal antibody against the E2 protein of GETV, developed in our laboratory (Li F. et al., 2022), was used as the primary antibody.

### 2.3 Immunofluorescence staining

Immunofluorescence (IF) assay was performed to detect GETV and LC3 expression. Treated paraffin sections or cells were incubated with primary antibodies overnight at 4°C, washed with a washing solution, incubated with secondary antibodies at room temperature in the dark for 1.5 h, and then incubated with DAPI at room temperature in the dark for 5 min. The primary antibodies used were GETV E2 (1:200, developed in our laboratory; Li F. et al., 2022) and LC3A/B (1:200, 12741S, Cell Signaling Technology) rabbit antibodies (Li et al., 2019). The secondary antibodies were Alexa Fluor 647-labeled Goat Anti-Rabbit IgG (H + L; 1:500, A0468, Beyotime) and Alexa Fluor 488-labeled Goat Anti-Rabbit IgG (H + L; 1,500, A0423, Beyotime). The cell nuclei were stained with DAPI (1,1,000; Biodragon).

### 2.4 Detection of oxidative stress levels

We measured the levels of malondialdehyde (MDA; S0131S, Beyotime) and superoxide dismutase (SOD; S0087, Beyotime) in testicular tissue lysates according to the manufacturer’s instructions.

The mouse testes were dissected into small pieces and incubated with 5% trypsin at 33°C for 30 min (Lee et al., 2019). After incubation, the tissues were washed with pre-chilled PBS and suspended in PBS to a final concentration of 10^6^ cells/ml. For TM3 cells in different experimental groups, the cells were also washed with pre-chilled PBS and suspended in PBS to 10^6^ cells/ml. In brief, 300 μl of the cell suspension was added to a 5 ml tube, followed by staining with 10 μM dihydroethidium (DHE; Beyotime, S0063) at 37°C for 30 min. After staining, the cells were washed with PBS and centrifuged at 600 × *g* for 5 min. The supernatant was discarded, and the cells were resuspended in 0.5 ml PBS. the mean fluorescence intensity was determined using a BD FACS Calibur flow cytometer (Guo et al., 2022).

For TM3 cells in different groups, cells were washed twice with PBS and incubated with 10 μM DHE for 30 min. After staining, the cells were washed again with PBS and fixed with 4% paraformaldehyde. Fluorescent images were captured using a fluorescence microscope.

### 2.5 Transmission electron microscopy

At 24 hpi, in accordance with the protocol for transmission electron microscopy, the following steps were taken. The GETV-infected cells were digested in a 60 mm culture dish using trypsin. After centrifugation at 1,000 rpm for 5 min, the supernatant was discarded, and the cells were resuspended in diluted fixative (3% glutaraldehyde:0.1 mol/L PB = 1:5) and allowed to stand at 4°C for 5 min. Following centrifugation at 12,000 rpm for 10 min, the supernatant was discarded, and 3% glutaraldehyde fixative was carefully added to fix the cell aggregates, which were then placed in a foam box with ice packs and transported to the Lilai Biomedicine Experiment Center (Chengdu, Sichuan) for transmission electron microscopy.

### 2.6 Cell viability assay

Cell viability was determined by the CCK-8 assay (Beyotime, C0037) as the manufacturer instructions. In the experiments, NAC, Rapa and 3-MA were added in cells 1 h before GETV infection (Chen et al., 2024). and the CCK-8 assay was performed at 24 h after GETV infection.

### 2.7 Western blotting

RIPA lysis buffer (Beyotime, P0013B) containing protease inhibitors (Beyotime, P1005-1), EDTA (Beyotime, P1005-2), and phosphatase inhibitors (Beyotime, P1081) was used for effective cell lysis. Subsequently, the protein concentration was determined using an Enhanced BCA Protein Assay Kit (Beyotime, P0010S). Protein samples were prepared with 5× SDS-PAGE loading buffer. SDS-polyacrylamide gel electrophoresis was performed with approximately 50 μg of protein, followed by the transfer of proteins to a PVDF membrane. The membrane was blocked with 5% skim milk and then incubated with the appropriate primary antibodies at 4°C overnight. LC3A/B (D3U4C) XP® Rabbit mAb (PA5-22731, 1:1000), GAPDH (D4C6R) Mouse mAb (97166S, 1:1000), and SQSTM1/p62 (D6M5X) Rabbit mAb (23214S, 1:1000) were obtained from Cell Signaling Technology. The Spectra™ Multicolor Broad Range Protein Ladder (26634) was obtained from Invitrogen (Thermo Fisher Scientific—CN). For protein detection, the membranes were incubated with Anti-rabbit IgG, HRP-linked Antibody (1:3000, 7074P2, Cell Signaling Technology) and Anti-mouse IgG, HRP-linked Antibody (1:3000, 7076P2, Cell Signaling Technology). Finally, the signals were visualized with SuperSignal™ West Pico Plus Chemiluminescent Substrate. The gray intensity of proteins was measured using ImageJ software.

### 2.8 Statistical analysis

All results are presented as mean values with standard error. Statistical analysis, including multiple comparisons, was performed using SPSS software (version 17.0). Statistical significance was defined as significant at *p* < 0.05.

## 3 Results

### 3.1 GETV induced oxidative stress and autophagy in mouse testis

**Figure 1:**
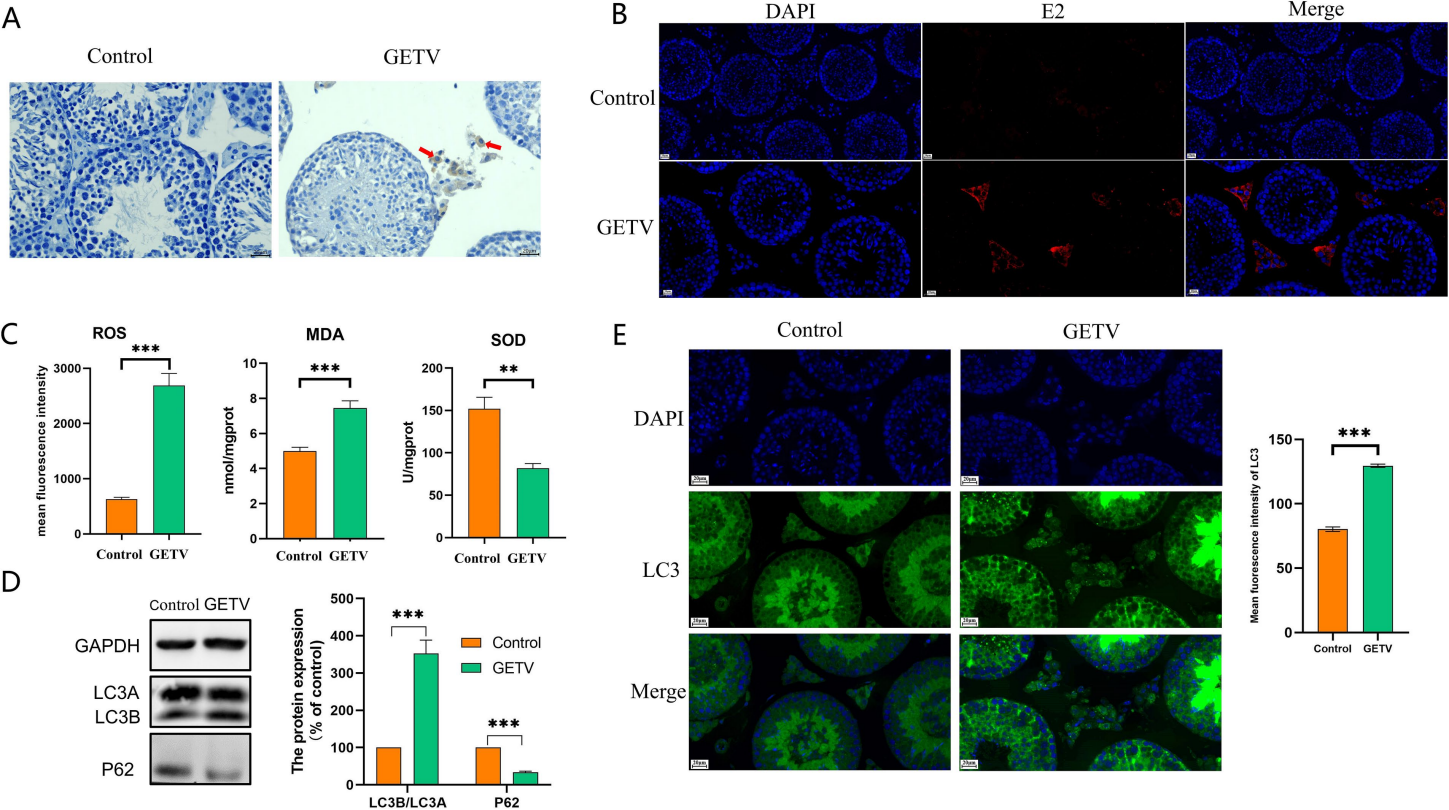
GETV induced oxidative stress and autophagy in the mouse testes. **(A)** The GETV E2 IHC staining of the testes shows that GETV-positive cells are found only in the testicular interstitium (indicated by red arrows). **(B)** The GETV E2 IF staining of the testes also shows that GETV-positive cells are found only in the testicular interstitium (the red cells). **(C)** Oxidative stress levels in mouse testes. ROS and MDA levels increase, while SOD level decreases in GETV group. **(D)** Expression of LC3A/B and P62 in the mouse testes. **(E)** Immunofluorescence images showing representative LC3 staining (green). Scale bars, 20 μm. *p ≤ 0.05 was considered statistically significant, while **p ≤ 0.01 and ***p ≤ 0.001 were deemed highly significant.

Using the GETV E2 protein as the primary antibody, the IHC and IF results indicated that GETV could infiltrate the mouse testis, with positive cells exclusively identified in Leydig cells (Figures 1A,B). To investigate the effect of GETV infection on oxidative stress levels in testicular tissues, ROS, MDA, and SOD levels were measured in testicular tissues. The results revealed that following GETV infection, the mean fluorescence intensity of ROS and MDA levels were significantly elevated, while SOD levels were notably reduced (Figure 1C), indicating that GETV infection caused an increase in oxidative stress levels in the testis. Western blot (WB) and IF analyses demonstrated that after GETV infection, the P62 protein levels in the mouse testis significantly decreased, while LC3B/A levels significantly increased (Figure 1D), alongside an elevation in LC3 fluorescence intensity (Figure 1E), indicating that GETV infection led to an increase in autophagy levels in the testis.

### 3.2 GETV infected TM3 cells *in vitro*

**Figure 2:**
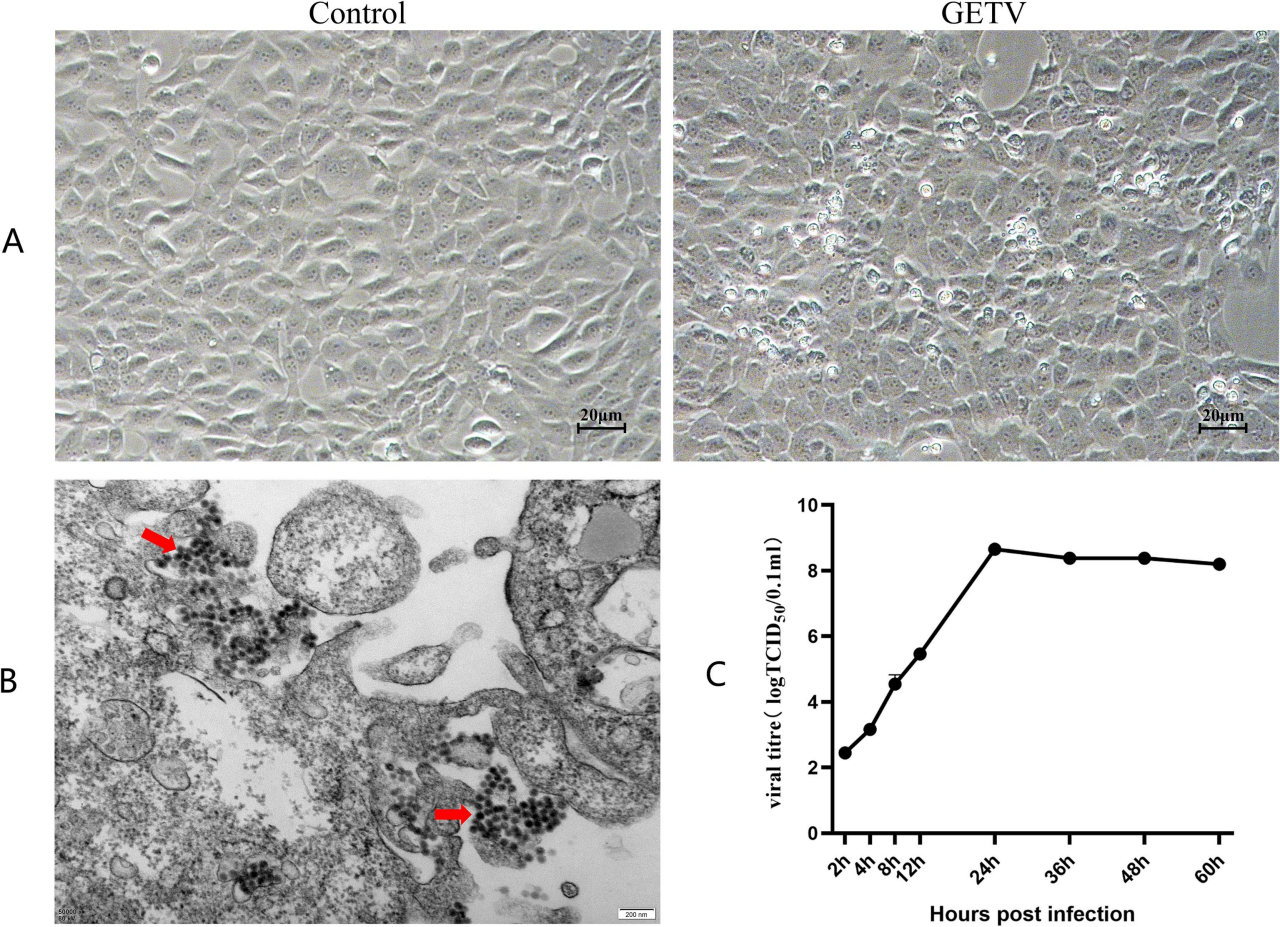
GETV Infection in TM3 Cells. **(A)** Cellular pathology following GETV infection. Scale bars, 20 μm. **(B)** Virus particles present in TM3 cells (indicated by red arrows). Scale bars, 200 nm. **(C)** Growth curve of GETV in TM3 Cells.

We found that GETV only infected interstitial cells in mouse testes. Hence, TM3 cells were the optimal in vitro model for research. TM3 cells were infected with 100 × TCID₅₀ in a total volume of 100 μl of GETV. The cells were examined under an inverted microscope, and images of cellular pathology were captured. After 12 h of infection, TM3 cells displayed cellular lesions characterized by shrinkage and rounding, and by 24 h, the cells started to detach (Figure 2A). Transmission electron microscopy (TEM) revealed virus particles with a diameter of approximately 70 nm, exhibiting a spherical shape, a membrane, and fibrous protrusions within the infected cells (Figure 2B), consistent with previous reports.

The supernatants collected at 2, 4, 8, 12, 24, 36, 48, and 60 h were used for the TCID₅₀ assay via the Reed-Muench method. The viral titer at 2 h was approximately 10^−3^.^32^ TCID₅₀/ml. Viral titers exhibited exponential growth during the initial 24 h. After 24 h, the rate of viral growth slowed down, reaching a plateau at approximately 36 h, and was maintained at approximately 10^−8^.^85^ TCID₅₀/ml (Figure 2C), which was consistent with the growth trend observed in other cells.

### 3.3 GETV induced oxidative stress and autophagy in TM3 cells

**Figure 3:**
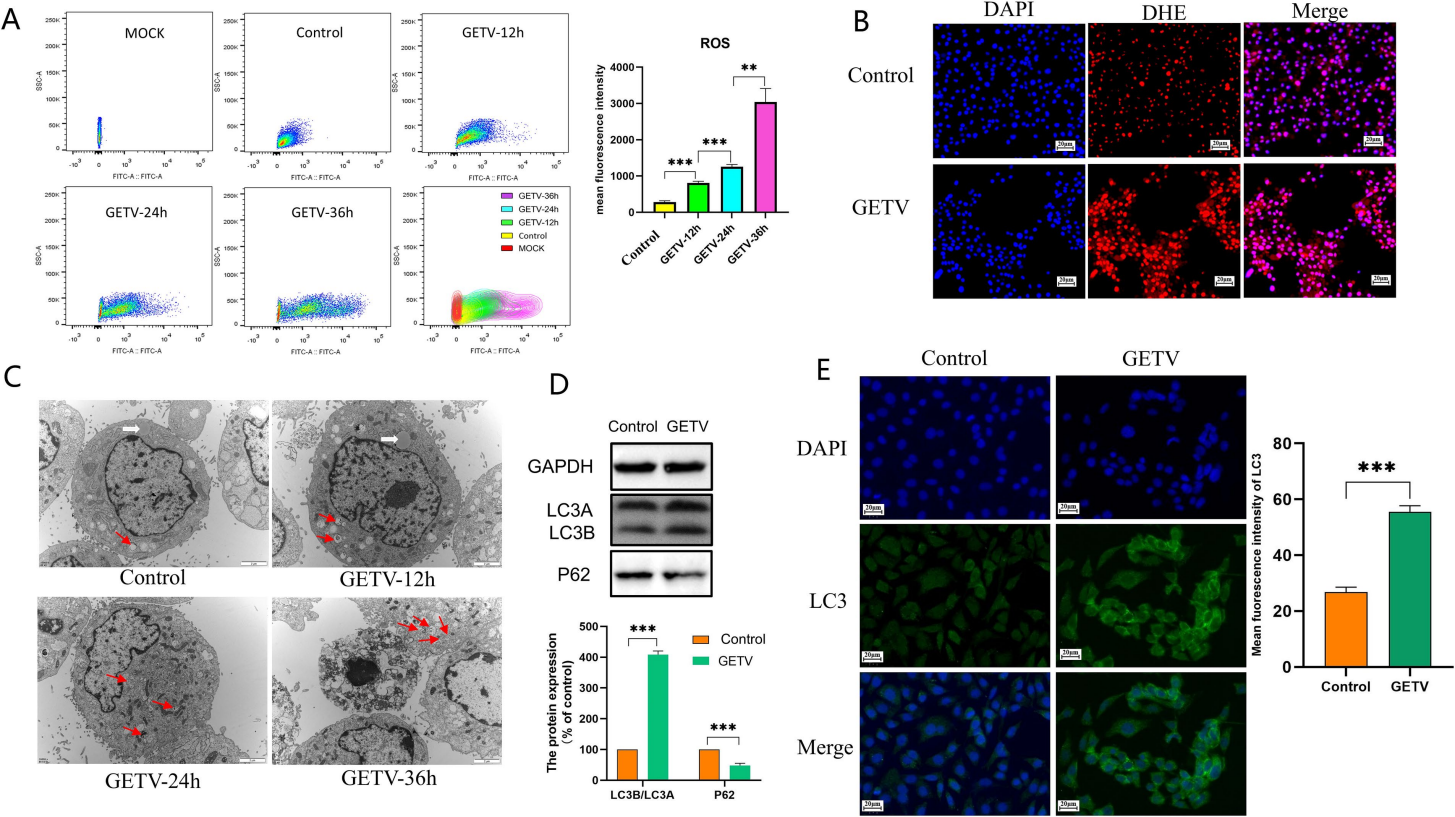
Effects of GETV Infection on Oxidative Stress and Autophagy in TM3 Cells. **(A)** ROS Levels in TM3 Cells. **(B)** The DHE IF staining of the TM3 Cells. Scale bars, 20 μm. **(C)** Presence of Autophagosomes in TM3 Cells (indicated by arrows). Scale bars, 2 μm. **(D)** Expression Levels of P62 and LC3B/A. **(E)** Immunofluorescence images showing representative LC3 staining (green). Scale bars, 20 μm. Statistical significance was deemed statistically significant at * *p* ≤ 0.05, while ** *p* ≤ 0.01 and *** *p* ≤ 0.001 were deemed highly significant.

As shown in Figure 3, after GETV infection, flow cytometry analysis indicated an increase in the mean fluorescence intensity of ROS (Figure 3A). IF detection revealed that the fluorescence intensity of ROS was elevated (Figure 3B). The number of autophagosomes increased (Figure 3C), the protein levels of P62 significantly decreased, while LC3B/A levels significantly increased (Figure 3D). The fluorescence intensity of LC3 increased (Figure 3E), suggesting that GETV infection resulted in elevated oxidative stress and enhanced autophagy in TM3 cells.

### 3.4 Inhibition of ROS reduces the autophagy level induced by GETV

To investigate the interaction between ROS and autophagy following GETV infection, we initially inhibited ROS production using NAC both *in vivo* and *in vitro*.

**Figure 4:**
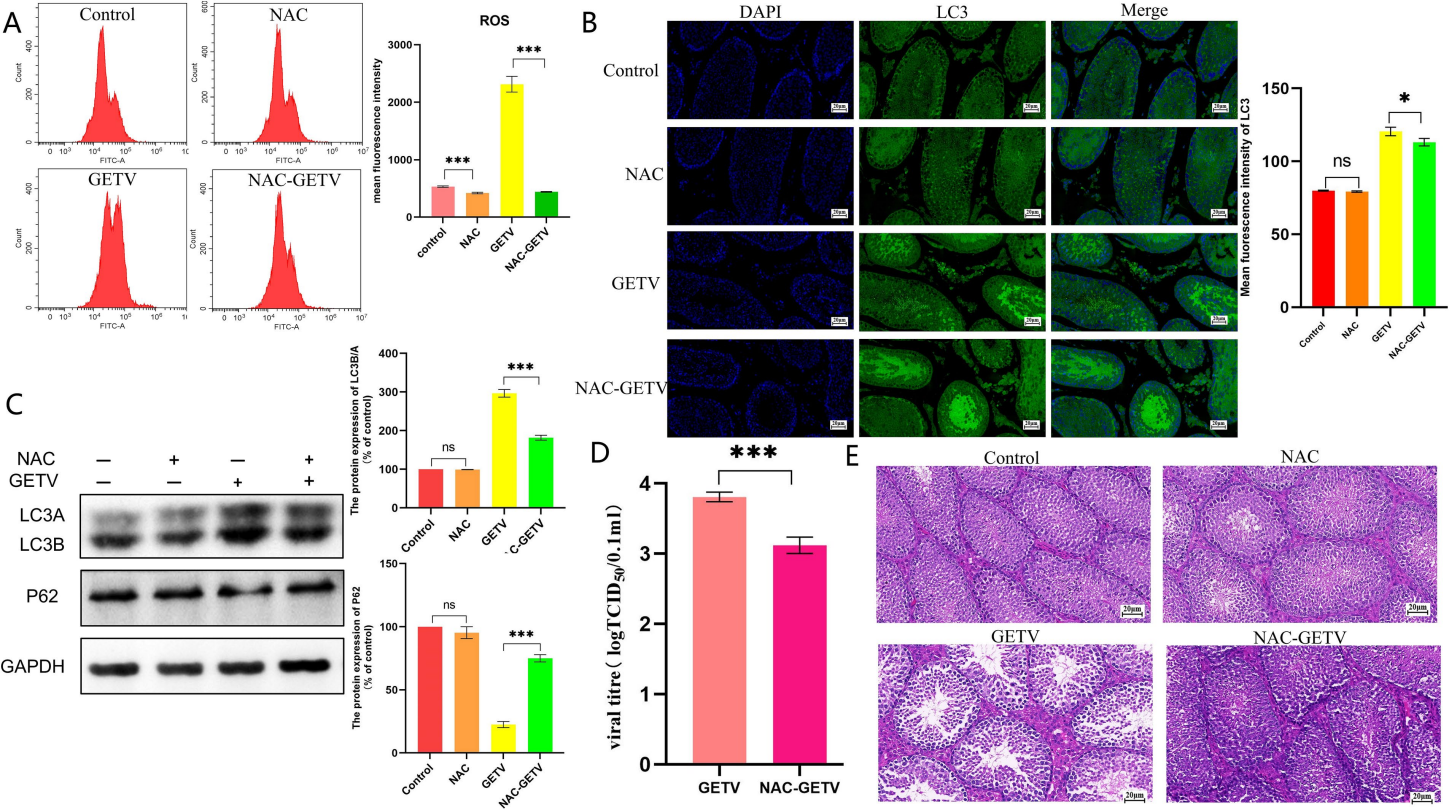
N-acetylcysteine (NAC) inhibits autophagy in testicular tissue through the inhibition of ROS production. **(A)** Flow cytometry analysis of reactive oxygen species (ROS) levels. **(B)** Immunofluorescence images showing representative LC3 staining (green). **(C)** Expression levels of P62 and LC3B/A. **(D)** TCID_50_ of GETV in testicular tissue. **(E)** Histological (H&E) staining of testes tissue section. Scale bars, 20 μm. * *p* ≤ 0.05 indicates a significant difference, while ** *p* ≤ 0.01 and *** *p* ≤ 0.001 indicate highly significant differences.

Flow cytometry was employed to assess the effect of NAC on ROS levels in mouse testes, revealing that NAC significantly reduced ROS levels in the NAC and NAC-GETV groups compared to the corresponding groups without NAC (Figure 4A).

Immunofluorescence results for LC3 indicated that both the GETV and NAC-GETV groups exhibited significantly enhanced green fluorescence compared to the control and NAC groups. In the GETV group, more intense fluorescence signals were observed in both the seminiferous tubules and interstitial tissue. Conversely, in the NAC-GETV group, the fluorescence signal was primarily localized to the seminiferous tubules (Figure 4B). WB results indicated that autophagy levels in the GETV and NAC-GETV groups were significantly higher than those in the control and NAC groups. However, the autophagy level in the NAC-GETV group was significantly lower than that in the GETV group (Figure 4C).

Additionally, we noted that the TCID_50_ of GETV in the testes of the NAC-GETV group was significantly lower than that in the GETV group (Figure 4D).

Observation of HE-stained testicular tissue sections revealed that NAC did not significantly alter the structure of mouse testicular tissue but effectively alleviated the injury caused by GETV. Compared to the GETV group, the NAC-GETV group exhibited more sperm in the seminiferous tubules, reduced damage to spermatogonia and spermatocytes, and less widening of the testicular interstitial space (Figure 4E).

**Figure 5:**
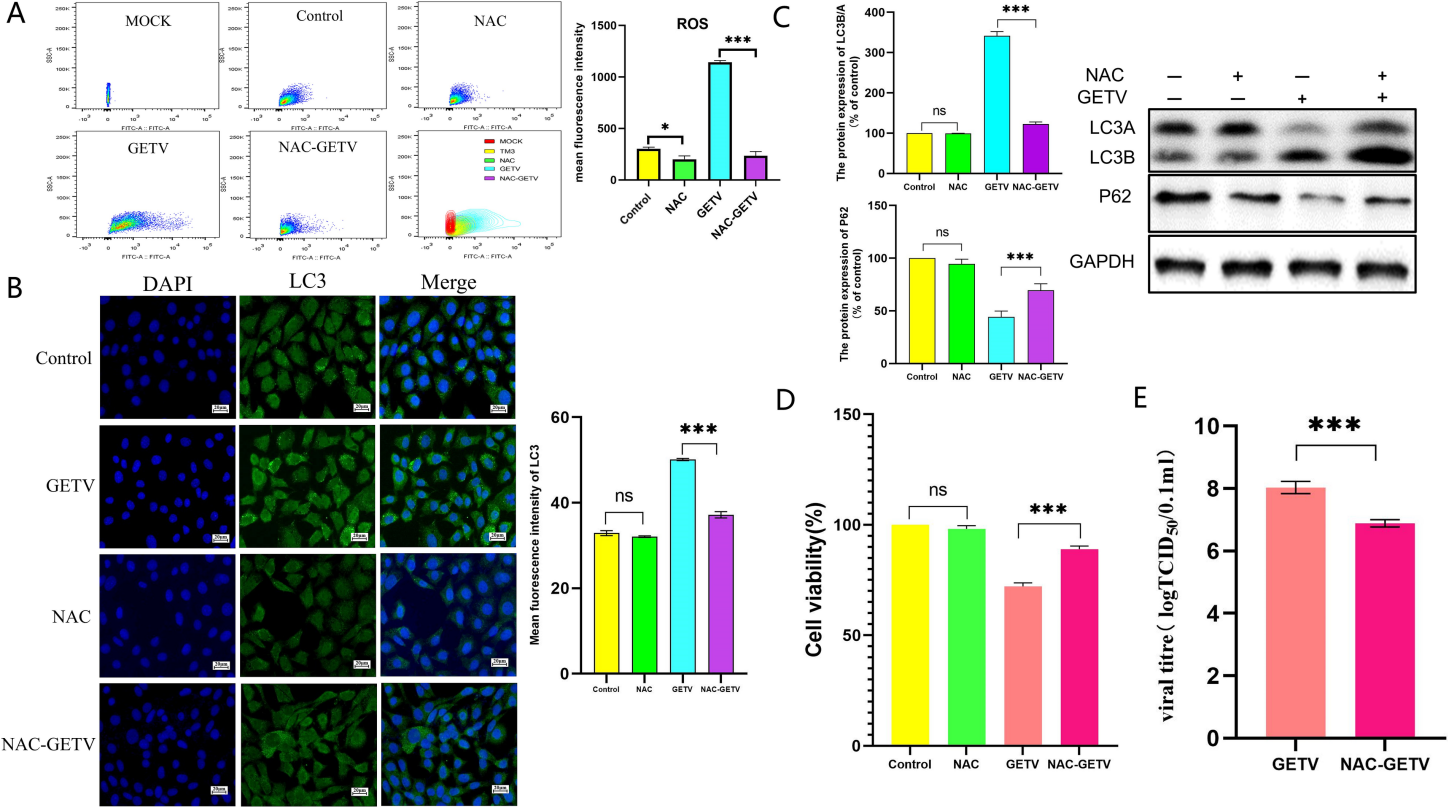
NAC inhibits autophagy in TM3 cells through the reduction of reactive oxygen species (ROS) production. **(A)** Flow cytometry analysis of ROS levels in cells. **(B)** Immunofluorescence images displaying representative staining of LC3 (green). **(C)** The expression levels of P62 and LC3B/A. Scale bars, 20 μm. **(D)** Cell viability across the experimental groups. **(E)** TCID_50_ of GETV in TM3 cells. * *p* ≤ 0.05 indicates a significant difference, while **p ≤ 0.01 and ***p ≤ 0.001 denote highly significant differences.

The results obtained from TM3 cells indicated that NAC significantly reduced ROS levels in both uninfected and GETV-infected cells (Figure 5A).

However, the reduction in ROS levels modulated the expression of autophagy-related proteins solely in GETV-infected cells, without significantly affecting autophagy in uninfected TM3 cells (Figures 5B,C).

Compared to the control group, NAC did not significantly alter the viability of TM3 cells, however, the viability of the NAC-GETV group was higher than that of the GETV group (Figure 5D). Additionally, the TCID_50_ of GETV in the NAC-GETV group was lower than that in the GETV group. (Figure 5E).

Both *in vivo* and *in vitro* results demonstrated that inhibiting ROS production in GETV-infected tissues and cells resulted in reduced autophagy levels, indicating that GETV-induced autophagy is regulated by ROS.

### 3.5 Autophagy did not change the ROS levels induced by GETV infection

To further investigate the interaction between ROS and autophagy following GETV infection, we subsequently interfered with autophagy levels using Rapa and 3-MA in TM3 cells.

**Figure 6:**
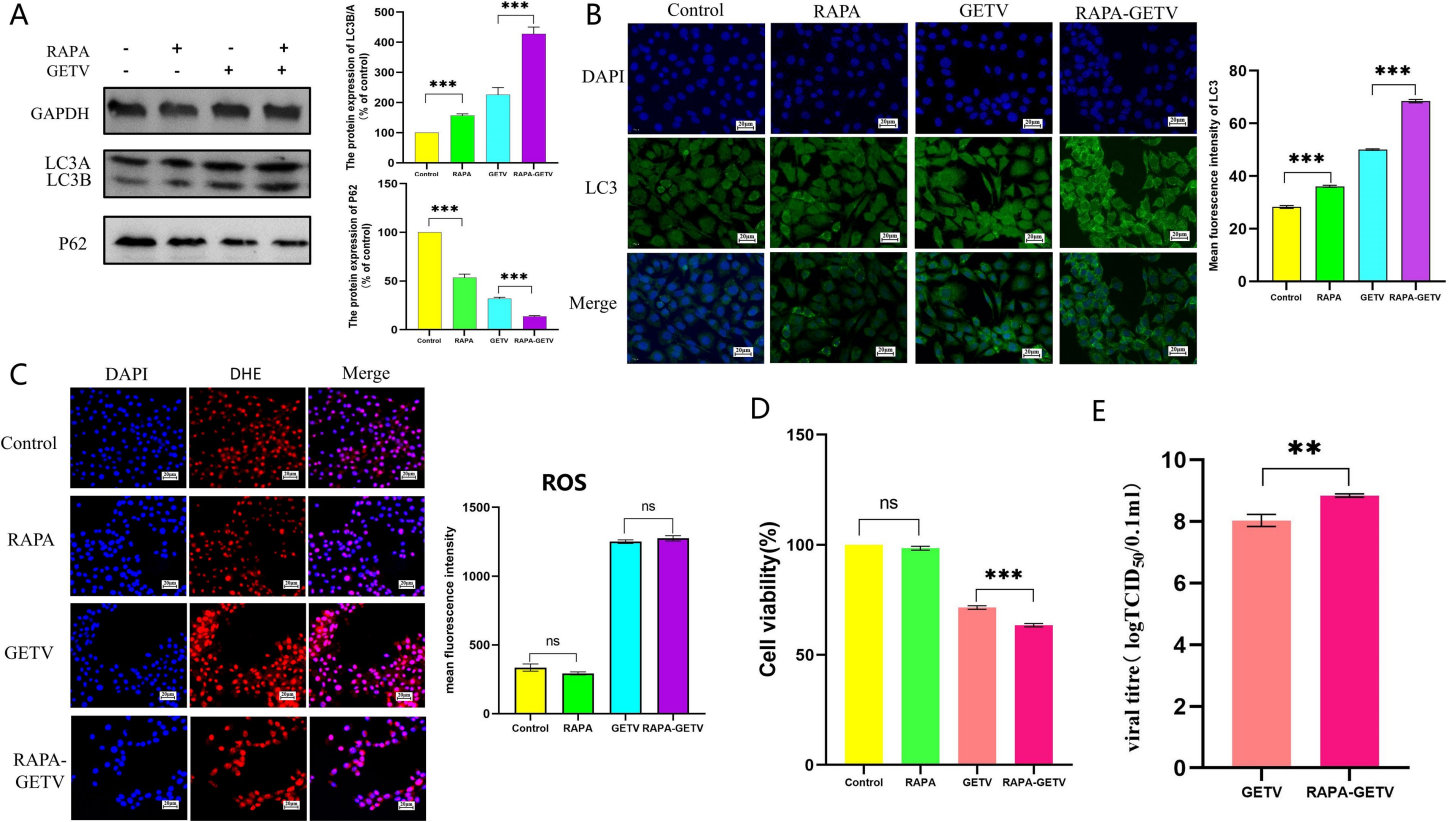
Effects of rapamycin (Rapa) on ROS Levels. **(A)** The expression levels of P62 and LC3B/A. **(B)** Immunofluorescence images displaying representative staining of LC3 (green). **(C)** The DHE IF staining of the TM3 Cells and the mean fluorescence intensity of ROS in TM3 Cells. **(D)** Cell Viability Across the Experimental Groups. **(E)** TCID_50_ of GETV in TM3 cells. Scale bars, 20 μm. * *p* ≤ 0.05 indicates a significant difference, while ** *p* ≤ 0.01 and *** *p* ≤ 0.001 denote highly significant differences.

The results indicated that Rapa significantly increased autophagy levels in both GETV-infected and uninfected TM3 cells (Figures 6A,B). Compared to the control group, ROS levels in the Rapa group were lower but not significantly different, while both the GETV and Rapa-GETV groups exhibited a significant increase, with no significant difference between these two groups (Figure 6C). This indicates that the ROS changes induced by GETV in TM3 cells are not influenced by autophagy levels. Compared to the control group and GETV group, cell viability in RAPA-GETV group was lower (Figure 6D). Additionally, the TCID_50_ of GETV in the RAPA-GETV group was higher than that in the GETV group (Figure 6E), suggesting that GETV can exploit autophagy to enhance its replication.

**Figure 7:**
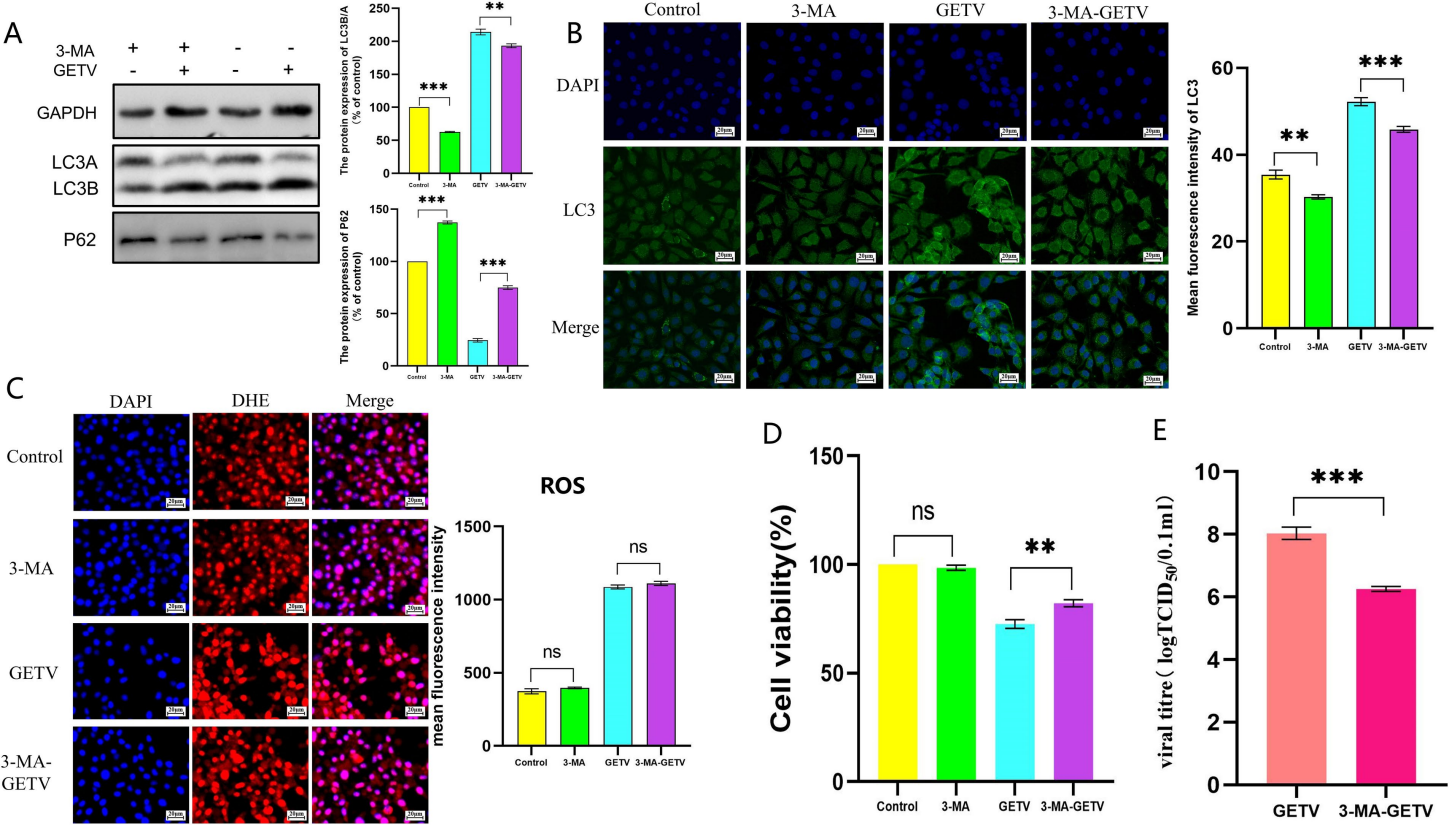
Effects of 3-methyladenine (3-MA) on ROS Levels. **(A)** The expression levels of P62 and LC3B/A. **(B)** Immunofluorescence images displaying representative staining of LC3 (green). **(B)** ROS Levels in TM3 Cells. **(C)** The DHE IF staining of the TM3 Cells and the mean fluorescence intensity of ROS in TM3 Cells. **(D)** Cell Viability Across the Experimental Groups. **(E)** TCID_50_ of GETV in TM3 cells. Scale bars, 20 μm. * *p* ≤ 0.05 indicates a significant difference, while ** *p* ≤ 0.01 and *** *p* ≤ 0.001 denote highly significant differences.

The experimental results of 3-MA further supported this hypothesis. Treatment with 3-MA significantly inhibited autophagy levels in TM3 cells (Figures 7A,B). However, 3-MA did not cause significant changes in ROS levels in either GETV-infected or uninfected TM3 cells (Figure 7C). Moreover, the use of 3-MA alleviated the impact of GETV replication on cell viability (Figure 7D), which might be related to the reduction in infectious viral particles of GETV in cells treated with 3-MA, as indicated by the decrease in TCID_50_ (Figure 7E).

## 4 Discussion

GETV is transmitted by mosquitoes and poses a potential risk to human health. Threats to the natural environment and public health should be addressed seriously, particularly considering the virus’s impact on male reproductive function, which raises concerns regarding animal welfare and the overall health of livestock populations (Li et al., 2017a). Leydig cells are located in the interstitium outside the seminiferous tubules and are crucial for steroidogenesis and spermatogenesis, serving as the primary source of male testosterone or androgens. These androgens diffuse from the interstitium into the seminiferous tubules and act on germ cells and supporting cells to induce and sustain sperm production (Park et al., 2023). In our previous studies, we found that after GETV infection, only Leydig cells in mouse testes were infected with GETV (Li F. et al., 2022). In this study, we used IHC and IF to validate these findings. Although GETV did not directly infect other cells in the testis, instead infecting only Leydig cells, the infection of Leydig cells led to changes in the testicular microenvironment, thereby greatly impacting the reproductive function of male mice (Zhou et al., 2019; Li H. et al., 2023). TM3 cells, derived from male mice aged 11 to 13 days, possess the physiological functions of Leydig cells under experimental conditions and are widely used in research (Zhao et al., 2022). Therefore, this study utilized TM3 cells as the cellular model for relevant *in vitro* studies of the impact of GETV on male reproductive function.

As products of oxidative stress, ROS not only participate in normal physiological and biochemical processes but also play a critical role in maintaining redox balance. Leydig cells generate intracellular ROS from various sources and are located in proximity to testicular macrophages (Chi et al., 2024), making them susceptible to extracellular sources of ROS (Hou et al., 2023; Huang et al., 2024). Many viral infections can cause changes in ROS levels, including influenza virus (Wang L. et al., 2022), chikungunya virus (Hiroki et al., 2019), and others (Rosa et al., 2023). Our study demonstrated that GETV infection induces elevated ROS levels in Leydig cells both *in vivo* and in vitro.

Autophagy is essential for maintaining the homeostasis and function of interstitial cells, including the regulation of steroidogenic activity (Dong et al., 2021). During viral infection, the host typically utilizes autophagy as a defense mechanism against the virus (Chen et al., 2023). However, many viruses have evolved strategies to disrupt this pathway and hijack autophagy components for their own benefit (Echavarria-Consuegra et al., 2019; Stoyanova et al., 2023). Studies have shown that the autophagy pathway promote DENV and West Nile virus (WNV) replication and release (Lee et al., 2008; Mateo et al., 2013; Blázquez et al., 2014). This study observed an increase in autophagy levels following GETV infection *in vitro* and *in vivo*. Notably, when autophagy levels were reduced with 3-MA, a corresponding decrease in TCID_50_ of GETV was observed. Conversely, upregulation of autophagy using Rapa resulted in an increase in TCID_50_ of GETV. Therefore, we speculated that GETV may exploit autophagy to enhance its replication capacity.

Studies have shown that ROS can influence autophagy (Kma and Baruah, 2022). Excessive ROS can disrupt this internal balance, ultimately leading to autophagic degradation or cellular apoptosis (Luo et al., 2019; Xing et al., 2022). We aimed to investigate whether there is a connection between ROS induced by GETV infection and autophagy. Our results indicated that GETV infection elevated ROS levels in testicular tissue and TM3 cells, resulting in enhanced autophagy. This finding aligns with observations from numerous other viruses. To further investigate the relationship between ROS and autophagy after GETV infection, GETV-infected male mice and TM3 cells were treated with NAC. The results indicated a reduction in ROS levels within the NAC and NAC-GETV groups, accompanied by an increase in P62 protein levels and a decrease in LC3B/A protein expression. Overall, GETV-induced autophagy is mediated by ROS. However, when TM3 cells were treated with Rapa and 3-MA, the ROS levels in the RAPA-GETV group and 3-MA group showed no significant change compared to the GETV group. These results indicated that GETV-induced ROS generation is probably regulated by mechanisms beyond the autophagy pathway (Piperis et al., 2024).

This study investigated the effects of GETV infection on oxidative stress and autophagy in mouse testes and TM3 cells. The results demonstrated that GETV infection leads to increased reactive oxygen species (ROS) levels, which mediate autophagy in mouse Leydig cells. We also found that autophagy plays an important role in GETV replication. Specifically, GETV may exploit the autophagy pathway to facilitate its replication. These findings illuminate the interplay between GETV and host cellular, providing valuable insights for the treatment strategy of GETV infection.

## Data Availability

The original contributions presented in the study are included in the article/Supplementary material, further inquiries can be directed to the corresponding author.
