# Supplementary figures and images for: Getah virus triggers ROS-mediated autophagy in mouse Leydig cells
Source: Front Microbiol. 2025 Jan 13;15:1519694. doi: 10.3389/fmicb.2024.1519694 (PMC11771000; doi:10.3389/fmicb.2024.1519694)

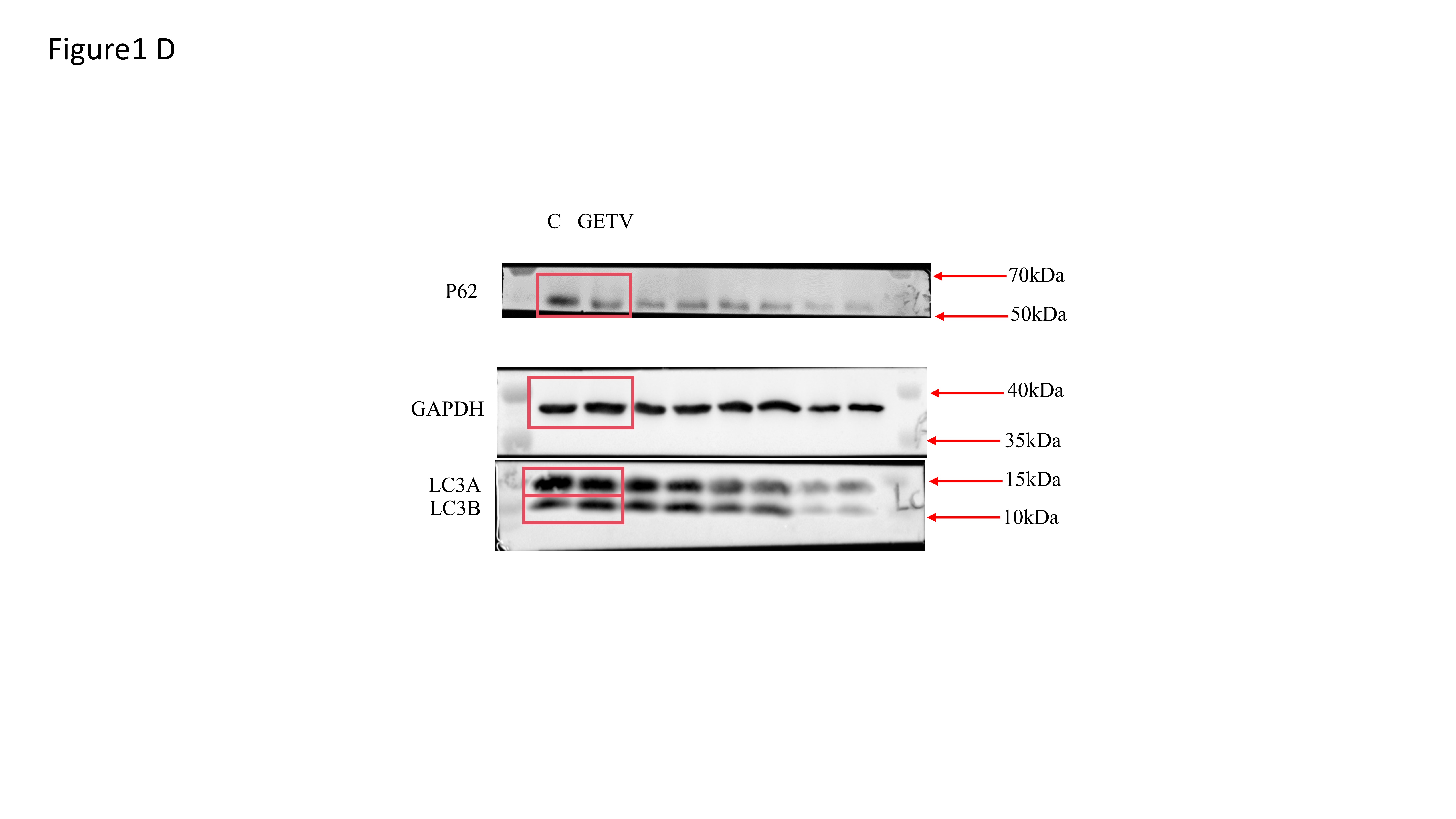

Supplement: Supplementary file 1 [file Image_1.jpg]

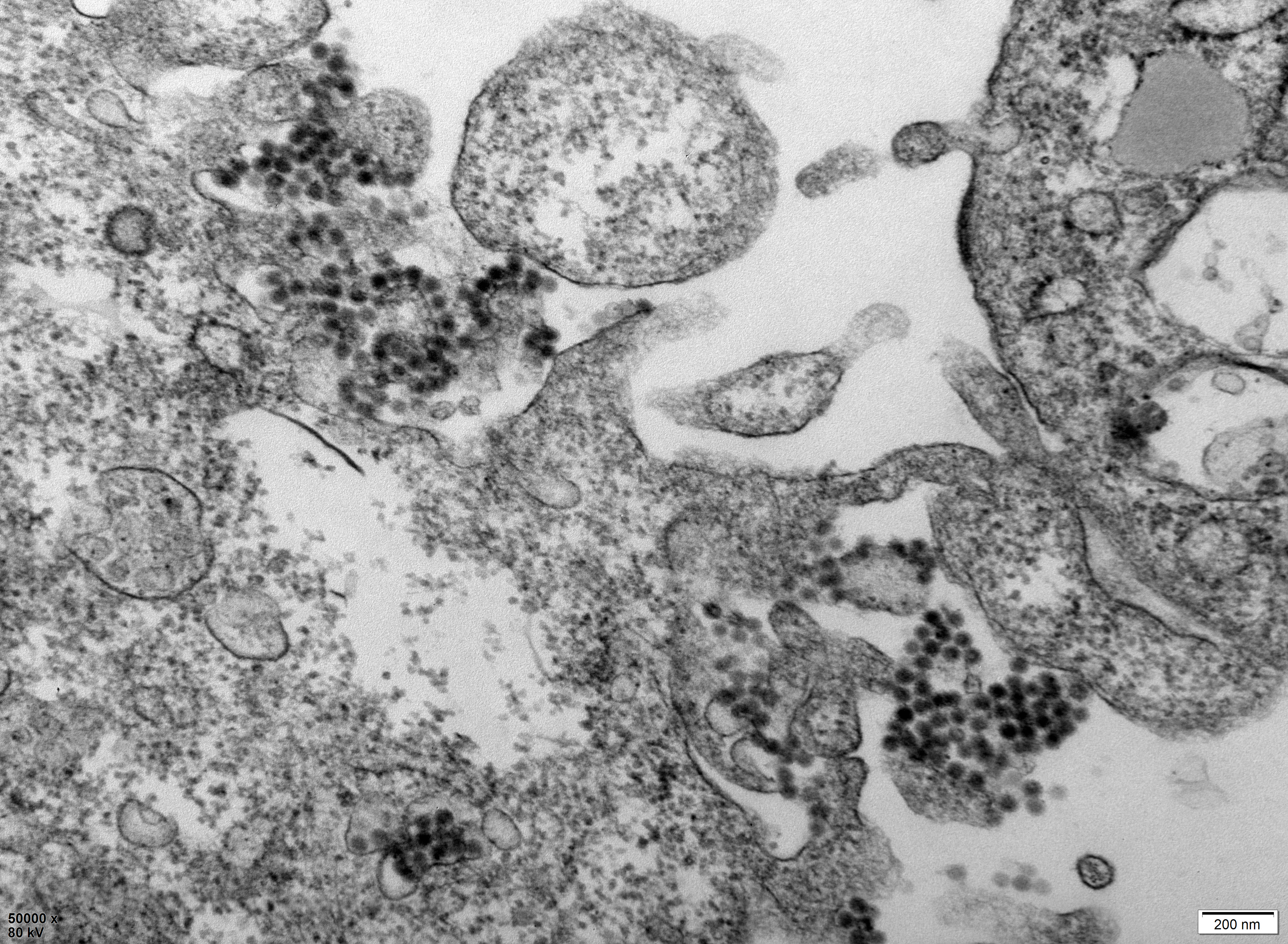

Supplement: Supplementary file 2 [file Image_2.tif]

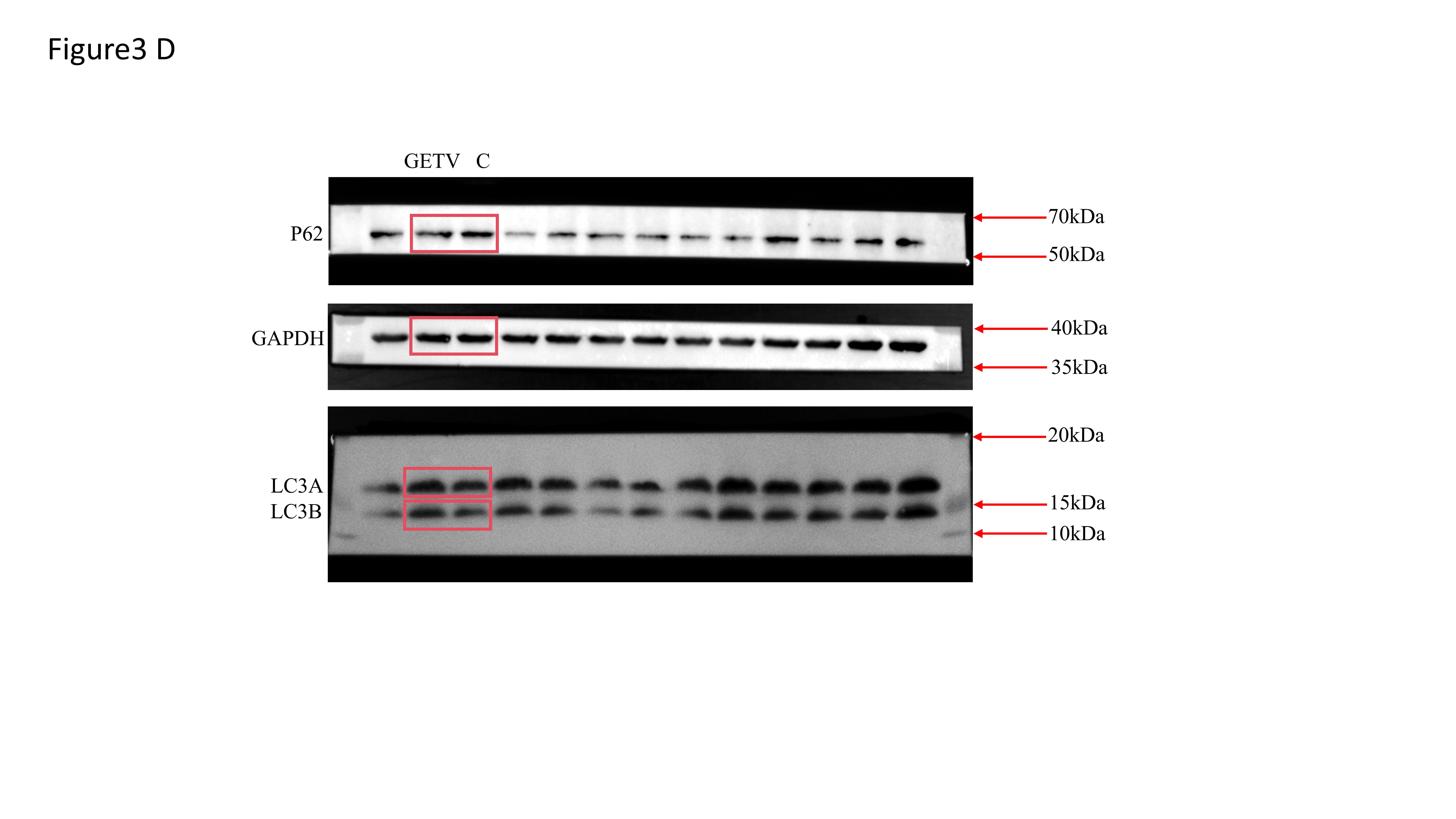

Supplement: Supplementary file 3 [file Image_3.jpg]

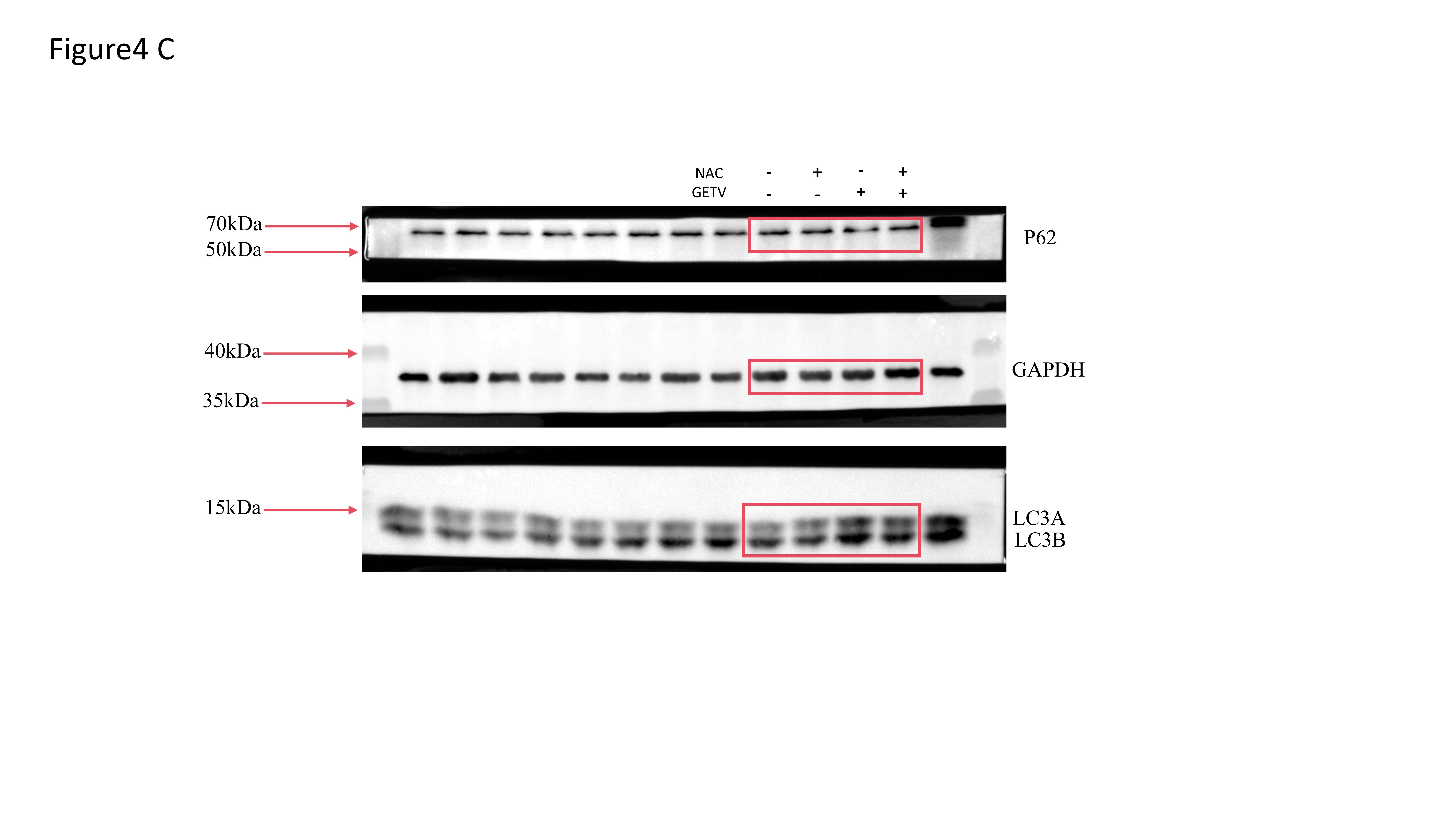

Supplement: Supplementary file 4 [file Image_4.jpg]

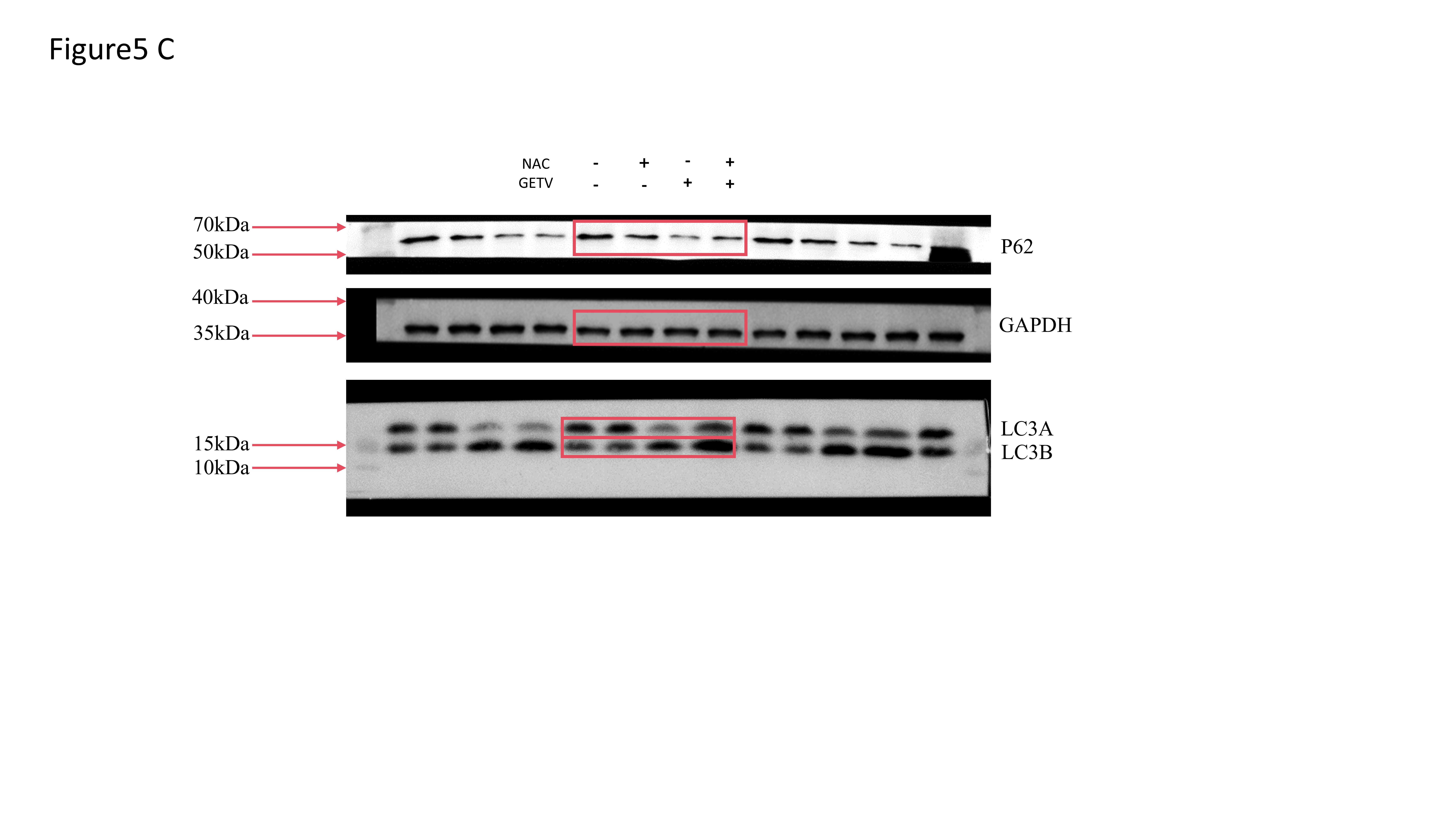

Supplement: Supplementary file 5 [file Image_5.jpg]

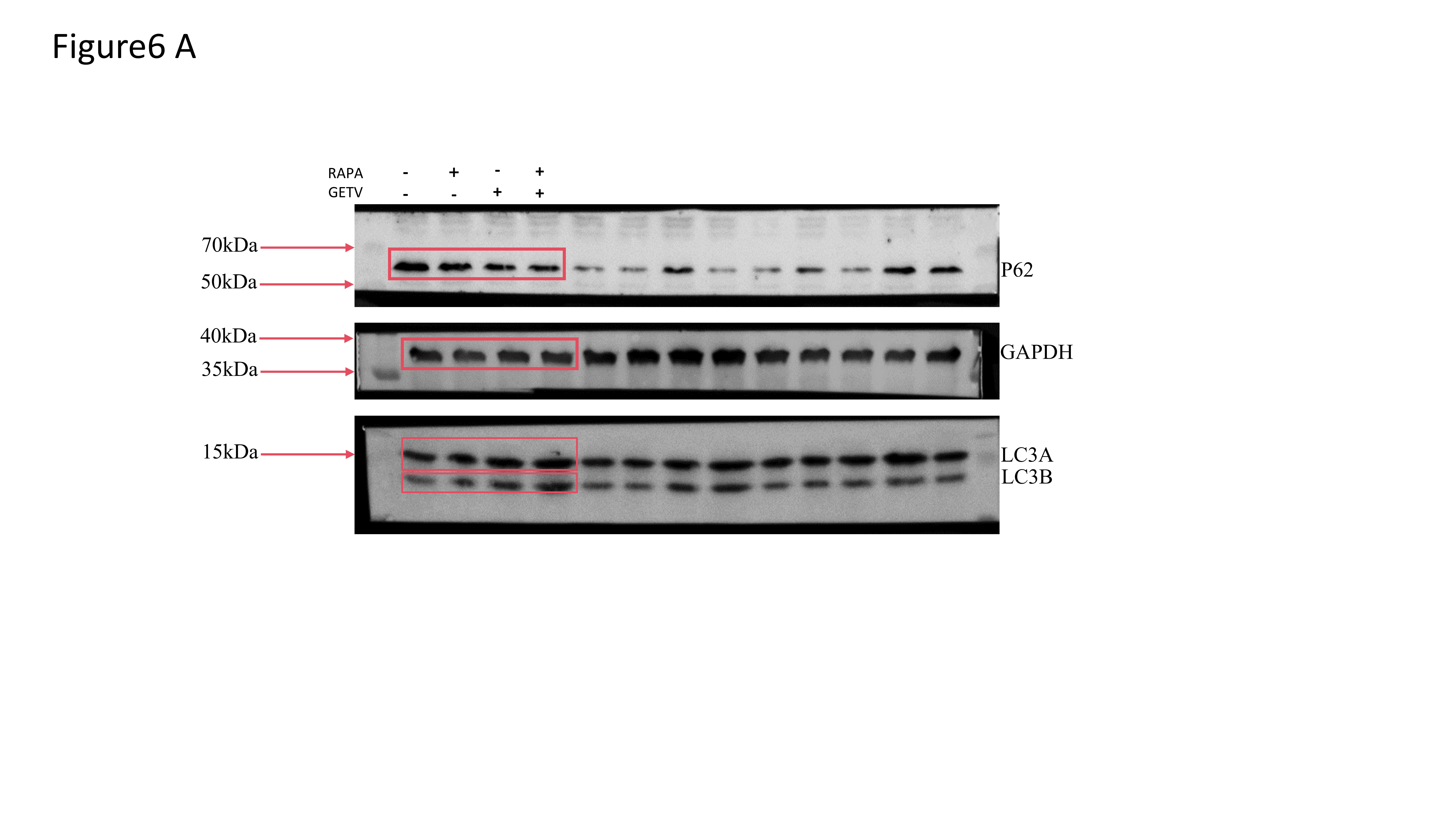

Supplement: Supplementary file 6 [file Image_6.jpg]

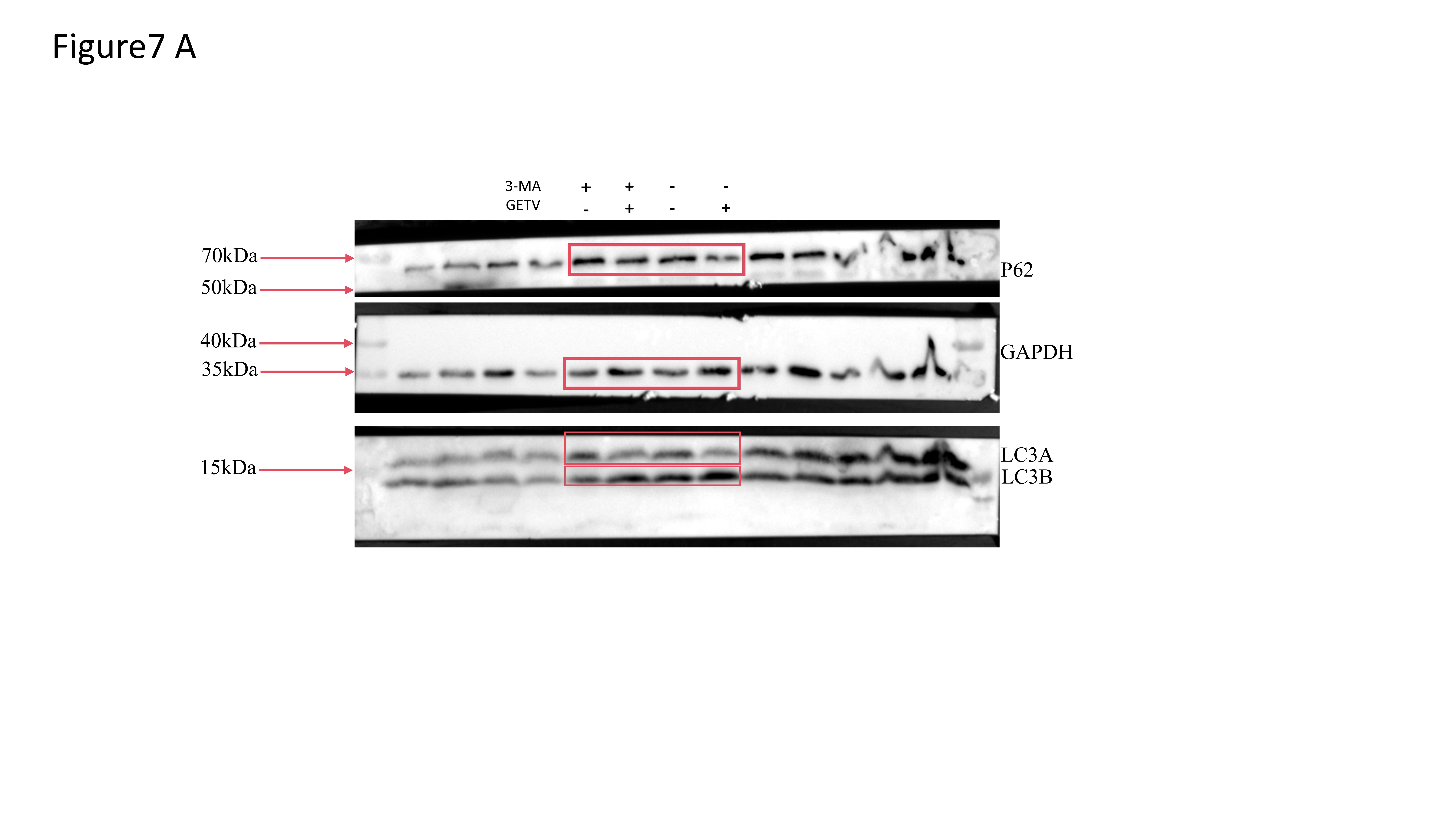

Supplement: Supplementary file 7 [file Image_7.jpg]
